# Spatio-Temporal Dynamics and Sensitive Distance Identification of Light Pollution in Protected Areas Based on Muti-Source Data: A Case Study of Guangdong Province, China

**DOI:** 10.3390/ijerph191912662

**Published:** 2022-10-03

**Authors:** Benyan Jiang, Shan Li, Jianjun Li, Yuli Zhang, Zihao Zheng

**Affiliations:** 1 College of Architecture and Urban Planning, Guangzhou University, Guangzhou 510006, China; 2 School of Geography and Remote Sensing, Guangzhou University, Guangzhou 510006, China

**Keywords:** protected areas, light pollution, sensitive distance, governance, Guangdong

## Abstract

Ecosystems in protected areas (PAs) are facing new challenges from light pollution. Timely assessment of light pollution in protected areas and exploration of its characteristics are positively valuable for biodiversity conservation and sustainable development. As the province with the largest number of nature reserves and the richest biodiversity in China, Guangdong’s protected areas have faced more severe light pollution threats in recent years along with rapid urbanization. Hence, in this study, the temporal trends and spatial distribution of light pollution changes, the correlation between light pollution and human activities, and the sensitive distance induced by light pollution in PAs of Guangdong Province from 2000 to 2018 were analyzed based on nighttime light (*NTL*) remote sensing data, land-use data, and POI data. The results show that: (1) Overall, the light pollution within the PAs increased significantly in these years, with the mean value of *NTL* (MNTL) increasing from 8.04 to 15.21 nanoWatt/cm^2^/sr. The number of PAs affected by light pollution was 900 in 2018, accounting for 83% of the total. (2) From the perspective of spatial distribution, the PAs in the Pearl River Delta suffered from the highest intensity of light pollution. Specifically, the MNTL of PAs within the Pearl River Delta was 5.8 times and 2.8 times higher than that in northern Guangdong in 2000 and 2018, respectively. (3) There is a significant correlation between human activities and *NTL* in PAs within 100 km, and the most sensitive distance is within 40 km, especially the sensitivity within 20 km is much higher than that between 30–40 km. The findings of this study will provide a reference for the implementation of external lighting control and guidance measures to mitigate light pollution in protected areas in Guangdong Province.

## 1. Introduction

Although the invention and widespread use of electricity is one of the most important human technological advances [1], the rapid global increase in artificial light at night (ALAN) has been widely considered a new threat to ecosystems [2,3,4]. Studies have shown that ALAN has profound impacts on the behavior of various organisms [3], leading to negative consequences for biological processes, biodiversity, and the functioning of ecosystems [5,6]. Moreover, almost all of the global ecosystems are experiencing the impacts of ALAN, whether in Asia, South America, or Africa [7,8,9], and whether terrestrial ecosystems or marine ecosystems [10,11,12]. Scholars worry that light pollution may reshape the global ecosystem [1].

In addition to ecological light pollution, astronomical light pollution has also raised concerns. It degrades the view of the night sky, which should be a precious natural property for all human beings. Nowadays, it is almost impossible for people to have a chance to gaze at the starry sky as more than 80% of the world, and especially 99% of the Americans and Europeans, live under an alight-polluted sky [13]. Repairing the dark night environment where the public can stargaze has become a new driving force for many countries to control light pollution.

Protected areas (PAs) are supposed to be the last refuges for both ecosystems and the night sky. However, PAs have also been widely invaded by light pollution. In Europe and North America, high proportions of individual protected areas (>17%) have exhibited high levels of nighttime lighting in all recent years [14], and in China, the light pollution in PAs increased by about 1.79 times from 1992 to 2012 [15]. It is estimated that ALAN is rapidly spreading globally at a rate of 6% per year [1], which means that PAs will probably be exposed to a higher risk of light pollution if no effective policies are instituted.

To enforce effective repair and preventative measures, it is critical to quantify the extent of nighttime lighting in PAs and its influential factors. Remote sensing nighttime light (*NTL*) data has become an important reference to monitor the *NTL* environment of PAs because it is macroscopic, rapid, and temporally continuous [16,17,18,19]. The spatio-temporal *NTL* data captured by satellite makes it possible for scholars to study evolution trends and spatial differences between PAs. For example, some scholars used remotely-sensed *NTL* data to analyze light pollution in China’s PAs between 1992 and 2012 [15,20], and proved that PAs in eastern China and provincial capitals are experiencing more serious light pollution [21,22]. Other studies have found that, on a global scale, coastal regions are experiencing an increasing *NTL* radiation as the economies of developing countries grow [10], with Mediterranean-climate ecosystems experiencing the greatest increases in exposure [5].

Having quantified temporal dynamics and spatial differences, scholars began to explore the reasons for the increase in light pollution. Infrastructure construction [8] and urban sprawl have been identified as the main sources of light pollution to PAs, with impact distance reaching approximately 245 km [9,13]. In previous studies, the most frequently used remote sensing data are products of the Defense Meteorological Satellite Program’s Operational Linescan System (DMSP/OLS) and Visible Infrared Imaging Radiometer Suite Day-Night Band (VIIRS/DNB), because they are easily accessible and cover *NTL* data from 1992 to the present [23].

Existing studies were conducted on a macro scale, such as the global scale, continental plate scale, and national scale, and they mainly attempted to identify the farthest distance that could be affected by ALAN. Few studies have been conducted on the spatial scale of urban planning. Therefore, this paper chooses the PAs in Guangdong, China as a study case, aiming to: (1) evaluate the spatio-temporal trends of *NTL* from 2000 to 2018 in the PAs of Guangdong and find out the correlation with human activities; (2) identify the sensitive threshold on an urban planning scale, within which the impacts of human disturbance are largest, and dark-repairing measures enforced within this distance would be most effective. It could be a reference to demarcate buffer zones of different grades and formulate corresponding light pollution repair strategies for PAs.

## 2. Materials and Methods

### 2.1. Study Area

**Figure 1:**
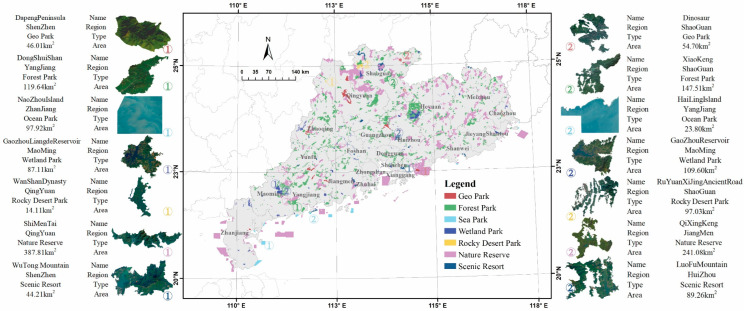
The spatial distribution of the PAs in Guangdong.

Guangdong Province is located in the southern part of mainland China, between 109°45′–117°20′ E and 20°09′–25°31′ N, with high terrain in the north and low terrain in the south. Guangdong established the first PA in China, and it is the only demonstration province for building PAs [24]. The number of PAs in Guangdong is 1085 [25], with a total area of 30,352 km^2^, accounting for about 14.3% of the terrestrial area of Guangdong (Figure 1). Meanwhile, Guangdong is the largest economic province of China with a population of about 127 million, which explains why its PAs have experienced the most severe light pollution [26].

The PAs in Guangdong are divided into seven types (Table 1), among which the Nature Reserve has the largest area of 15,586 km2, accounting for half of the PA areas. It is followed by the Forest Park with an area of 11,555 km2, whose number is as large as 617, ranking first among the seven types. There are also 10 sea parks located near or off the coastline, whose topography differs significantly from the terrestrial PAs. Considering the comprehensiveness of the samples, all the PAs designated by nation, province, city, and prefecture in Guangdong were included in this study. The vector boundaries of PAs were collected from the Guangdong Forestry Bureau.

### 2.2. Data Sources

Three main types of data have been used in this paper, summarized in Table 2.

#### 2.2.1. Nighttime Light Data

Remote sensing *NTL* data is generated by capturing faint light emitted by human activities on the ground, enabling direct measurements of upward light emission from the Earth’s surface without contacting ecologically sensitive areas [27]. As mentioned above, the most widely and frequently used *NTL* datasets are the products of DMSP/OLS and VIIRS/DNB. The DMSP/OLS *NTL* archive covers a long period from 1992 to 2012, providing annual synthetic *NTL* data, while monthly synthetic *NTL* data is available from the VIIRS DNB product from 2013 to the present DMSP, which consists of two broad spectral bands, a visible near-infrared band (0.4–1.1 µm) and a thermal infrared band (10.5–12.6 µm) [28]. It can detect the *NTL* radiance from m 1.54 × 10^−9^ to 3.17 × 10^−7^ W·cm^−2^·sr^−1^·µm^−1^ [29]. The VIIRS onboard the Suomi National Polar Partnership (NPP) was designed to collect high-quality radiometric data [29]. It can detect a specific dynamic range of approximately 7 orders of magnitude from 3 × 10^−9^ to 2 × 10^−2^ W·cm^−2^·sr^−1^, with a noise floor at about 5 × 10^−11^ W·cm^−2^·sr^−1^ [30,31,32].

Despite the distinct differences in sensors and calibrations between the two *NTL* datasets, some scholars have proposed methods for aligning heterogenous *NTL* images [33,34,35] and several post-processing products are freely available online [36,37]. In this paper, we used the harmonized global nighttime light dataset calibrated by Li et al. [36], spanning from 2000 to 2018.

#### 2.2.2. Human Activity Intensity Data

Two sources of datasets were used as main indicators in this study to characterize human activity intensity: land-use data and point of interest (POI) data of Guangdong. Specifically: (1) the land-use dataset of Guangdong was extracted based on the first Landsat-derived annual China land cover dataset conducted by Yang and Huang [38], which with higher spatial resolution and longer temporal coverage with regard to the existing annual land cover products. Its spatial resolution is 30 m and the overall accuracy reached 79.31 % under the post-processing method incorporating spatial–temporal filtering and logical reasoning, spanning from 1985 to 2020; (2) the POI dataset was provided by Amap. It includes 19 types: food services, health care, government agencies, residential services, financial insurance, etc. These two types of datasets were normalized and added with equal weights to generate the human activity intensity layer.

### 2.3. Methods

#### 2.3.1. Spatio-Temporal Changes of *NTL*

In this paper, three indicators, the sum of light intensity (TSOL), the mean *NTL* intensity (MNTL), and the proportion of lighted areas (POLA) were used to evaluate the *NTL* level with the PAs in Guangdong. TSOL is the sum of the pixel values for *NTL* radiation, MNTL is the mean value of *NTL* radiation, and POLA is the proportion of pixels with a digital number larger than 1. Besides, the Theil–Sen Median trend analysis and Mann–Kendall test were used to examine the *NTL* trends of the PAs in Guangdong, both of which are widely used tools for analyzing trends in vegetation, and atmospheric temperature [39,40]. The formula of Theil–Sen trend analysis is:

(1) $$\beta =Median\left(\frac{NTL_{j}-NTL_{i}}{j-i}\right),2000\leq i\leq j\leq 2018$$

where *NTL_j_* and *NTL_i_* are *NTL* radiation values at year *i* and *j*, respectively. When *β* > 0, it means the light intensity is increasing at the pixel; otherwise, it is decreasing.

The Mann–Kendall test is calculated as:

(2) $$Z=\left\{\begin{array}{c} \frac{S-1}{Var\left(S\right)},S>0\\ \;\;0,\;\;\;\;\;S>0\\ \frac{S+1}{\sqrt{Var\left(S\right)}},S<0 \end{array}\right.$$

(3) $$S=\sum_{i=1}^{n-1}\sum_{j=i+1}^{n}sgn\left(NTL_{j}-NTL_{i}\right)\;$$

(4) $$sgn\left(NTL_{j}-NTL_{i}\right)=\left\{\begin{array}{c} 1,NTL_{j}-NTL_{i}>0\\ 0,NTL_{j}-NTL_{i}=0\\ -1,NTL_{j}-NTL_{i}<0 \end{array}\right.$$

(5) $$Var\left(s\right)=\frac{n\left(n-1\right)\left(2n+5\right)}{18}$$

where sgn is the symbolic function; *n* is the length of the time series; *Z* is the significance test statistic with a value range of (−∞, +∞). At a confidence level of α = 0.05, $\left|Z\right|\;\geq \;Z_{\frac{0.05}{2}}=1.96$ indicates that there is a significant change in the *NTL* intensity.

#### 2.3.2. Correlation Detection

Due to the halo effect of light, the light pollution within one PA is in fact a cumulative result of the ALAN outside. Exploring the relationship between human activities outside PAs and light pollution within PAs can help us better optimize PA planning strategies to minimize environmental distance [9].

The following fitted regression model was used to explore the relationship between the *NTL* within PAs and human activity intensity:

(6) $$Y_{i,t}=\beta_{0}+K\times X_{i,d,t}+\varepsilon_{i,d,t}$$

where Y represents the MNTL dataset of the studied 1085 PAs, X represents the total value of human activity intensity in the d km buffer zone outside PAs; t represents the study year; K is the regression coefficient; $\varepsilon_{i,d,t}$ represents the error term.

The *NTL* data is freely available from remotely sensed data and previous studies. Considering that the spatial resolution of VIIRS DNB is about 15 arc seconds [32,41], much higher than DMSP. Therefore, the *NTL* data from VIIRS were used to calculate the MNTL as Y. As for human activity intensity, the equal-weighted value of land-use data and POI data was used as an indicator for X. The correlation between Y and X was examined through statistical analysis of 1085 PA samples. The coefficient K reveals whether they are positively or negatively correlated, and the correlation degree of X and Y.

To ensure the rationality of the results, two periods of 2013 and 2017 were extracted to validate the correlation between MNTL and human activity intensity in PAs. One point needed to be mentioned is that due to the data limitation, the POI data in 2012 was used as an alternative to that of 2013.

#### 2.3.3. Sensitive Distance Analysis

We made a hypothesis that there exists a sensitive distance within which the NLT in PAs has the greatest correlation with human activity intensity, which means the human activity within this distance should be most limited or guided. Therefore, this is a significant distance for PA protection planning.

**Figure 2:**
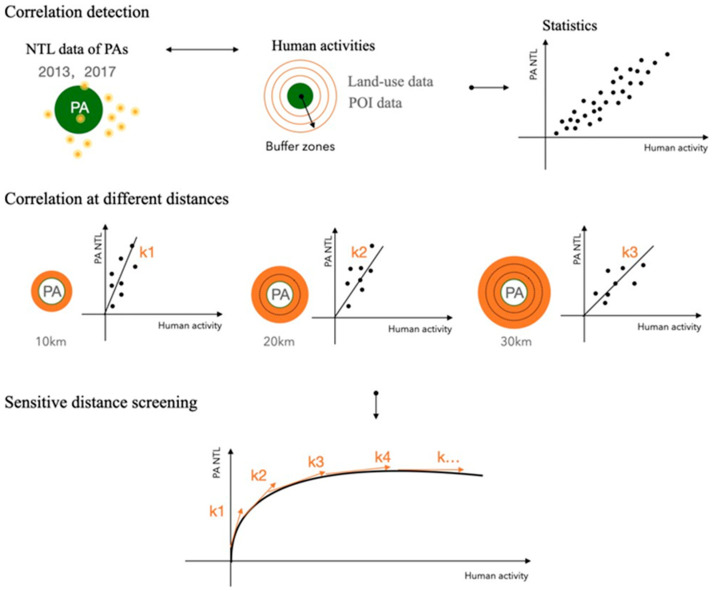
Research methods diagram.

The confirmation of sensitive distance is based on the results of correlation analysis in the last step. We hypothesize that human activity intensity at different distances has a different correlation degree with MNTL in PAs, hence the coefficient K is supposed to fluctuate in some range. The distance above which the coefficient tends to be stable will be our assumed sensitive distance (Figure 2).

## 3. Results

### 3.1. Temporal Trend of NTL within PAs

**Figure 3:**
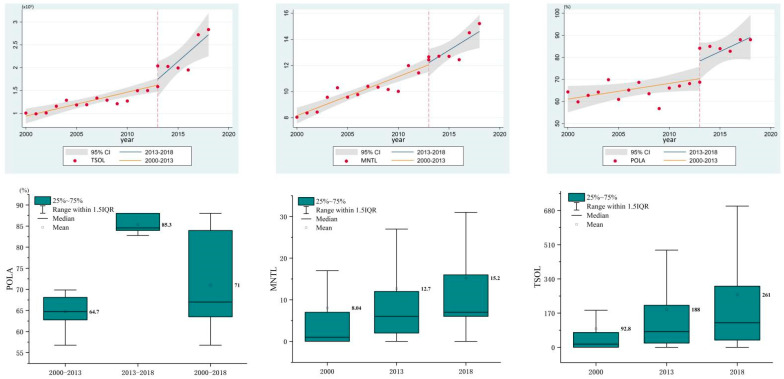
Statistics of *NTL* changes in PAs of Guangdong.

Figure 3 shows the temporal trends of *NTL* within the PAs from 2000–2018. In general, PAs in Guangdong have experienced a dramatic increase in *NTL* in the past 19 years, with TSOL jumping from 99,046 to 165,040, MNTL increasing from 8.04 to 15.21, and POLA expanding from 56.8% to 88.1%, indicating that only about 12% of the PA areas are unaffected by light pollution at the pixel level.

At the PA level, we calculated the MNTL annually for each PA because it better reflects the *NTL* degree [22]. The results in Table 3 show that 900 PAs experienced an *NTL* increase, accounting for 83% of the total study samples. By contrast, only 29 PAs showed the opposite decreasing trend, making up about 2.7%. In addition, there were 156 PAs with no obvious change in MNTL. Among the 900 PAs with increased MNTLs, there were 319 PAs free of light pollution in 2000 and experienced primary light pollution in the following 19 years, while 581 PAs experienced “secondary pollution” from 2000 to 2018.

In Figure 3, the orange fitting line represents the temporal trend between 2000 and 2012, and the blue fitting line represents the temporal trend between 2013 and 2018. The results clearly show that both the values and the growth rate of TSOL, MNTL, and POLA after 2013 are much bigger than those before 2013, especially for the TSOL result. These “jumping” results were caused by the use of different types of satellites that VIIRS is more sensitive and has higher spatial resolution than that of DMSP [29], which allows it to capture a larger amount of *NTL*.

### 3.2. Spatial Dynamics of NTL within PAs

**Figure 4:**
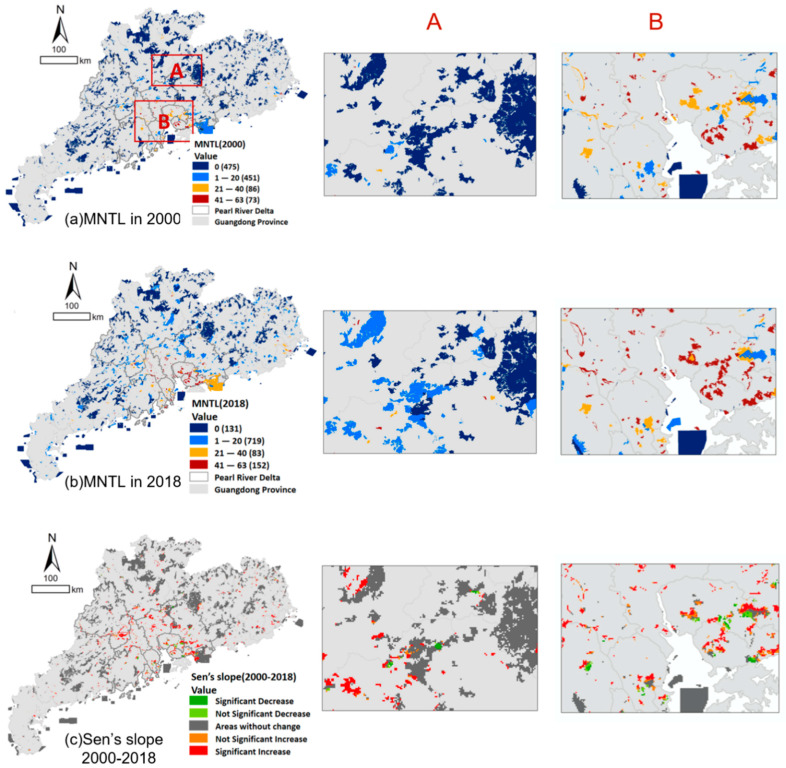
Spatial distribution and dynamics of MNTL.

In 2000, PAs exposed to severer light pollution were mainly concentrated in the Pearl River Delta and the eastern coast of Guangdong. The mean value of MNTL in this area was 15.94, and the maximum MNTL was 62. While in northern Guangdong, the maximum MNTL of PAs was only 2.74, and there were 235 PAs whose MNTLs were 0, explaining that they were little affected by light pollution in 2000. Detailed results are shown in Figure 4a.

In order to exclude the influence of satellite accuracy itself on the growth trend analysis, this study used the difference between the light value of VIIRS in 2013 minus that of DMSP as the gap between the two satellites, and then corrected the light value between 2013 to 2018 by VIIRS. In 2018, PAs with high MNTL were still concentrated in the Pearl River Delta and eastern coast, and the mean MNTL shifted to 24.82, 1.6 times that of 2000. Although the max MNTL was still 62 in 2018, the number of PAs with max MNTL jumped to 12, six times that of 2000. As for PAs in northern Guangdong, which had little light pollution in 2000 but experienced a serious *NTL* increase during these years, their MNTL increased from 2.74 to 8.92, and their maximum MNTL increased from 50 to 61, which was almost the same as that of PAs in the Pearl River Delta. The PAs with 0 MNTL decreased from 235 to 20, indicating that in the past 19 years, Pas in northern Guangdong experienced more widespread impacts from light pollution in the past years. Detailed results are shown in Figure 4b.

From Sen’s slope and MK significance analysis results shown in Figure 4c, we found that from 2000 to 2018, PAs that experienced a significant increase in MNTL were mainly concentrated in the Pearl River Delta as well. It is assumed that there are mainly two reasons for this result: first, the Pearl River Delta is the most economically developed region in Guangdong Province and even in China, with an urbanization level of 86% [42] and a population of 100 million according to the seventh census. While the urbanization level in northern Guangdong was only about 51%, with a population of 16,100 thousand [42]. Second, the flat terrain of the Pearl River Delta is more favorable for the haloing of *NTL*, while the mountainous area of northern Guangdong can block the transmission of *NTL*.

### 3.3. Correlation and Sensitive Distance Analysis

**Figure 5:**
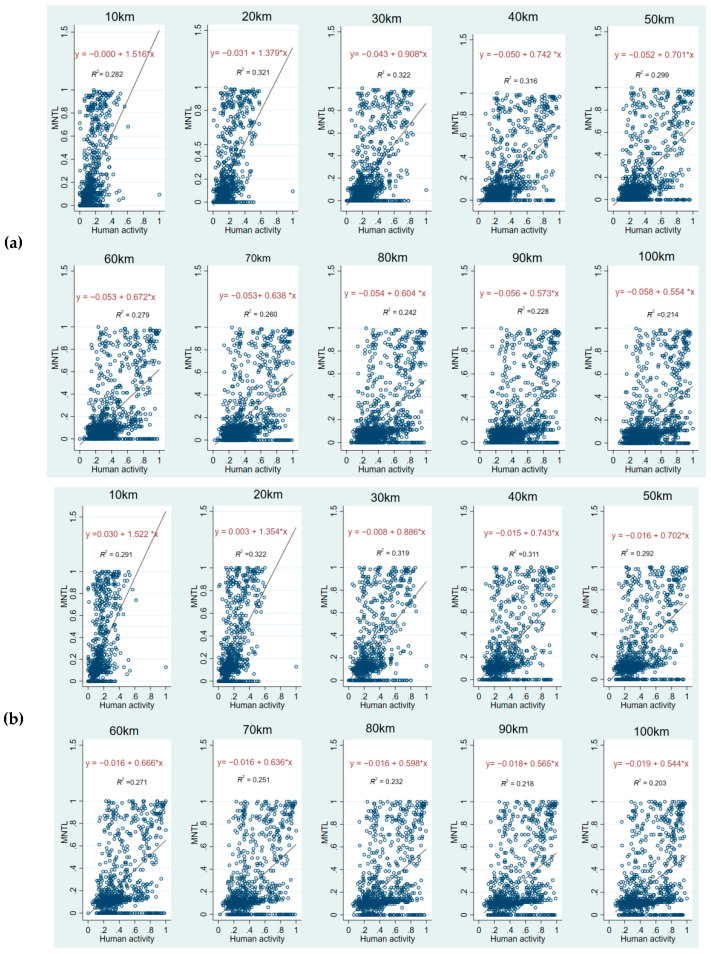
Scatter diagram of MNTL in PAs and human activity at different distances. (**a**) Fitting results in 2013; (**b**) Fitting results in 2017.

We divided 100 km outside the PA boundary into 10 gradients, calculated the mean values of human activity intensity at different buffer zones, and plotted the scatter plots of human activity intensity and the MNTL of PAs to reveal the correlation between them. The results of the correlation exploration are shown in Figure 5. The top panel (Figure 5a) shows in 2013 and the bottom panel (Figure 5b) shows in 2017. The key information in Figure 5 has been consolidated into Table 4.

According to the fitting results of 20 scatter plots in 2013 and 2017, an obvious conclusion is that the MNTL within the PAs and human activities within 100 km are positively correlated, that is, the PAs will face more serious light pollution if the human activity intensity outside rises. Despite the positive correlation, the degree of correlation between them varies at different distances. The coefficient of the fitting results could explain the degree of correlation between them, which provides a basis for us to screen out the sensitive distance.

As shown in Table 3, the correlation coefficient is greatest at 10 km and then decreases with increasing distance, which occurred in both 2013 and 2017. Comparing the coefficient values at different distances, it can be found that the variation is obvious within 40 km and stabilized beyond 40 km. This indicates that the *NTL* in PAs is sensitive to the variation of human activity intensity within 40 km, but not beyond 40 km. In 2013, the regression model coefficients at 20 km and 30 km were 1.379 and 0.908, respectively, with a difference of 0.471, which was much higher than the difference of 0.137 between 10 km and 20 km and 0.166 between 30 km and 40 km. This suggests that the *NTL* within PAs is most sensitive to the variations of human activity intensity within 20 km. In addition, the R-squared of the model was about 0.32 at 20 km, 30 km, and 40 km, which also further indicated that the model explained better at these three distances.

In terms of the sensitivity to human activity intensity at different distances, it showed an almost identical trend in 2017 as in 2013. Consequently, based on the verification of the calculation results in 2017, we can conclude that 40 km is the sensitive distance of *NTL* within PAs to human activities outside, and the sensitivity of the first 20 km is much higher than that of the last 20 km.

## 4. Discussion

### 4.1. Set up External NTL Governance Zones

Protected areas are considered to be the principal means of stemming the loss of biodiversity. The number of PAs across the world has seen a double growth over the last two decades [26,43,44,45]. Once a PA is established, human production and construction activities inside it will be restricted to different degrees in different protected zones [46]. Studies have shown that zoning is an effective approach for PA management, and those zones with more stringent protection are subject to less human disturbance [47,48].

Current protection measures mainly focus on the impacts of human activities inside PAs, and they were made based on the previous understanding that the biodiversity within protected areas can be well preserved by limiting human activities inside PAs. However, more and more studies have proved that the impact of human activities outside PA is much greater when it comes to light pollution. As mentioned earlier, several studies have already shown that the impact distance of *NTL* can be more than 200 km, which is beyond our common perception. What is more, our study results of the past 19 years clearly showed that the areas and intensity of light pollution increased with urbanization, which means if we do not pay enough attention to it, the threats from light pollution in PAs will probably be further aggravated in the future.

**Figure 6:**
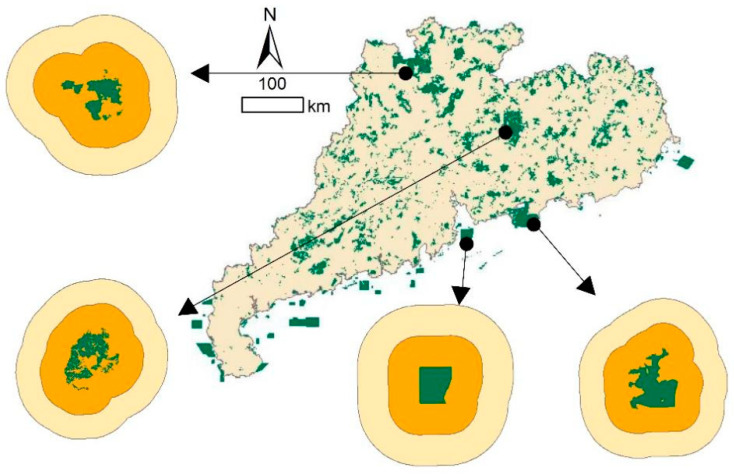
Light governance areas outside PAs. The inner orange zone is within 20 km, and the outer yellow zone is 20–40 km.

Therefore, it is necessary to discuss the feasibility of hierarchical control for ALAN outside PAs as with zoning inside PAs. Our study results identified that the most sensitive distance of *NTL* is 20 km, then 30–40 km. Therefore, these two areas can be demarcated as the core zone and buffer zone of *NTL* controls, respectively (Figure 6). The distance of 40 km is usually under the control of the smallest administrative unit, so it provides a scientific reference for planning and guiding artificial nighttime lighting from the urban planning level.

### 4.2. Adopt Differentiated Protection Measures

From the *NTL* spatial distribution results from 2000–2018, we found that although the values and intensity of *NTL* kept increasing in the 19 years, the spatial distribution shows obvious differentiation. In the case of Guangdong, PAs in the Pearl River Delta, the other coastal areas and the inland areas showed a clear difference. This kind of uneven spatial distribution phenomenon has high similarity at both national and global scales, because each region has different advantages, and the national or provincial orientations for them vary. It suggests that we may need to adopt corresponding conservation and repair strategies for PAs located in different areas.

In Guangdong Province, the Pearl River Delta is the engine of economic and industrial development. According to the Territorial Planning of Guangdong Province 2020–2035, industries will gather further in the future, and the urbanization level will increase to 90 percent by 2035. By contrast, the northern area of Guangdong is rich in ecological resources and has a low level of urbanization, and it is positioned as the ecological barrier of Guangdong province, whose urbanization level will be 65% by 2035, far lower than the Pearl River Delta city cluster. These two different development positions can provide guidance for us to formulate different *NTL* planning and night sky repairing strategies.

Considering the industrial development and people’s living needs for PAs in the Pearl River Delta, we have three suggestions to protect and repair the nighttime light environment. Firstly, reduce repeated lighting, because 30–60% of lighting consumption is unnecessary in cities [49]. Second, replace unsheltered light fixtures with sheltered ones, and this will effectively reduce the amount of direct upward light, which destroys the starry sky landscape. Third, shorten lighting time if it is possible, especially for those human activities that occur within the sensitive zones.

For PAs located in northern Guangdong, we propose to adopt more restrictive protection measures. It can be considered to designate a lighting control area at a distance of 40 km outside the boundary of a PA. Within this area, only the most necessary and minimal light can be used, such as road lighting. At a farther distance, *NTL* should also be planned and guided, measures mentioned above can be applied here as well.

### 4.3. Limitation and Further Study

This study carried out a long-term statistical analysis for the whole 1085 PAs in Guangdong based on the remotely sensed *NTL* data. Although the results contribute to the understanding of the increased light pollution in PAs, the spatial distribution of *NTL* and regional differences between PAs, and the sensitive distance of external *NTL*, there are still some aspects deserving further study. First, our study is totally based on satellite *NTL* data and no empirical study was conducted on individual PA to verify our results. Typical PAs with different types in different locations will be taken as cases to conduct field research and examine our findings in the next study. Second, our study came to a conclusion of sensitive disturbance distance, which needs a mechanistic analysis. Third, we proposed night sky protection and repair measures, and the effectiveness of each suggestion should be quantified through field measurements in further studies.

## 5. Conclusions

In this study, we carried out spatio-temporal dynamics of nighttime light within protected areas on a provincial scale from 2000–2018 based on calibrated joint data of DMSP/OLS and VIIRS/DNB. Our study results clearly confirmed the increasing temporal trends of light pollution within PAs, with the MNTL increasing from about 8 to 15, and about 83% of PAs suffering from changed nightlight environments. From the spatial distribution, PAs located in the Pearl River Delta experienced much higher and longer light pollution than other areas, because the urbanization level of this area is the highest in Guangdong. On the other hand, although PAs in northern Guangdong have lower MNTL, they have experienced more widespread impacts from *NTL* in numbers and areas.

We also explored the relationship between the *NTL* of PAs and human activities, aiming to make suggestions for decreasing light pollution and repairing the dark night sky. In general, *NTL* in PAs is positively correlated to the intensity of human activities within 100 km, and is especially sensitive to it within 40 km. Therefore, protection measures such as *NTL* governance areas, *NTL* plan, and guidance should be enforced within this distance. Finally, this paper quantifies the severity of *NTL* changes in PAs and it is hoped to promote the protection of the *NTL* environment and the construction of dark-night parks.

## Figures and Tables

**Table 1:** Properties of the PAs in Guangdong.

PA Types	Number	Proportion (%)	Area (km^2^)	Area Proportion (%)
Geo Park	14	1.3	835	2.8
Scenic Resort	22	2.0	710	2.3
Sea Park	12	1.1	530	1.7
Forest Park	611	56.3	11,155	36.8
Wetland Park	172	15.9	1408	4.6
Rocky Desert Park	3	0.3	128	0.4
Nature Reserve	251	23.1	15,586	51.4
Total	1085	100.0	30,351	100.0

**Table 2:** Data used in this study and their sources.

Dataset	Spatial Resolution	Temporal Resolution	Data Available	Access Link
*NTL* data	30 arc-seconds (around 1000 m)	Annual	2000–2018	https://figshare.com/articles/dataset/Harmonization_of_DMSP_and_VIIRS_nighttime_light_data_from_1992-2018_at_the_global_scale/9828827/2 (accessed on 8 August 2022) [36]
Land-use data	30 m	Annual	2013, 2017	https://zenodo.org/record/5210928#.YtgZf8g17qE (accessed on 8 August 2022) [38]
POI data	Vector data	Annual	2012, 2017	Amap

**Table 3:** Statistics of *NTL* changes in PAs of Guangdong.

Dynamics		Count		Proportion (%)	
*NTL* decrease			29		2.67
*NTL* with no change			156		14.38
*NTL* increase	Primary pollution	319	900	29.4	82.95
	Secondary pollution	581		53.55	

**Table 4:** Correlation between *NTL* in PAs and human activities at different distances.

(a) Regression Results of 2013										
	10 km	20 km	30 km	40 km	50 km	60 km	70 km	80 km	90 km	100 km
	Model (1)	Model (2)	Model (3)	Model (4)	Model (5)	Model (6)	Model (7)	Model (8)	Model (9)	Model (10)
Human activity	1.52 ** (0.073)	1.38 ** (0.061)	0.91 ** (0.040)	0.74 ** (0.033)	0.70 ** (0.033)	0.67 ** (0.033)	0.64 ** (0.033)	0.60 ** (0.032)	0.57 ** (0.032)	0.55 ** (0.032)
Constant	−0.000 (0.012)	−0.031 * (0.012)	−0.043 ** (0.013)	−0.050 ** (0.013)	−0.052 ** (0.014)	−0.053 ** (0.014)	−0.053 ** (0.015)	−0.054 ** (0.015)	−0.056 ** (0.016)	−0.058 ** (0.017)
Observations	1085	1085	1085	1085	1085	1085	1085	1085	1085	1085
R^2^	0.28	0.32	0.32	0.32	0.30	0.28	0.26	0.24	0.23	0.21
(b) Regression results of 2017										
	10 km	20 km	30 km	40 km	50 km	60 km	70 km	80 km	90 km	100 km
	Model (1)	Model (2)	Model (3)	Model (4)	Model (5)	Model (6)	Model (7)	Model (8)	Model (9)	Model (10)
Human activity	1.52 ** (0.072)	1.35 ** (0.060)	0.89 ** (0.039)	0.74 ** (0.034)	0.70 ** (0.033)	0.67 ** (0.033)	0.64 ** (0.033)	0.60 ** (0.033)	0.57 ** (0.033)	0.54 ** (0.033)
Constant	0.030 * (0.012)	0.003 (0.012)	−0.008 (0.013)	−0.015 (0.013)	−0.016 (0.014)	−0.016 (0.014)	−0.016 (0.015)	−0.016 (0.016)	−0.018 (0.016)	−0.019 (0.017)
Observations	1085	1085	1085	1085	1085	1085	1085	1085	1085	1085
R^2^	0.29	0.32	0.32	0.31	0.29	0.27	0.25	0.23	0.22	0.20

Notes: Standard errors in parentheses: ** *p* < 0.01, * *p* < 0.05.

## Data Availability

Not applicable.
